# Both hMutSα and hMutSß DNA Mismatch Repair Complexes Participate in 5-Fluorouracil Cytotoxicity

**DOI:** 10.1371/journal.pone.0028117

**Published:** 2011-12-02

**Authors:** Akihiro Tajima, Moriya Iwaizumi, Stephanie Tseng-Rogenski, Betty L. Cabrera, John M. Carethers

**Affiliations:** 1 Division of Gastroenterology, Department of Internal Medicine, University of Michigan, Ann Arbor, Michigan, United States of America; 2 Division of Gastroenterology, Department of Medicine, University of California San Diego, La Jolla, California, United States of America; 3 Moores Comprehensive Cancer Center, University of California San Diego, La Jolla, California, United States of America; Howard University, United States of America

## Abstract

**Background:**

Patients with advanced microsatellite unstable colorectal cancers do not show a survival benefit from 5-fluorouracil (5-FU)-based chemotherapy. We and others have shown that the DNA mismatch repair (MMR) complex hMutSα binds 5-FU incorporated into DNA. Although hMutSß is known to interact with interstrand crosslinks (ICLs) induced by drugs such as cisplatin and psoralen, it has not been demonstrated to interact with 5-FU incorporated into DNA. Our aim was to examine if hMutSß plays a role in 5-FU recognition.

**Methods:**

We compared the normalized growth of 5-FU treated cells containing either or both mismatch repair complexes using MTT and clonogenic assays. We utilized oligonucleotides containing 5-FU and purified baculovirus-synthesized hMutSα and hMutSß in electromobility shift assays (EMSA) and further analyzed binding using surface plasmon resonance.

**Results:**

MTT and clonogenic assays after 5-FU treatment demonstrated the most cytotoxicity in cells with both hMutSα and hMutSß, intermediate cytotoxicity in cells with hMutSα alone, and the least cytotoxicity in cells with hMutSß alone, hMutSß binds 5-FU-modified DNA, but its relative binding is less than the binding of 5-FU-modified DNA by hMutSα.

**Conclusion:**

Cytotoxicity induced by 5-FU is dependent on intact DNA MMR, with relative cell death correlating directly with hMutSα and/or hMutSß 5-FU binding ability (hMutSα>hMutSß). The MMR complexes provide a hierarchical chemosensitivity for 5-FU cell death, and may have implications for treatment of patients with certain MMR-deficient tumors.

## Introduction

The fluoropyrimidine 5-fluorouracil (5-FU) is the cornerstone for chemotherapy in patients with advanced stage colorectal cancer [1]. In addition to stage, the DNA mismatch repair (MMR) status of a patient's tumor appears to predict a survival response to 5-FU [2]. Patients with Lynch syndrome (germline mutation in MMR gene) or patients with sporadic microsatellite unstable (MSI) cancers (hypermethylation of the MMR gene *hMLH1*) do not show a survival advantage with systemic 5-FU therapy [3], [4], [5], [6], whereas patients with MMR-proficient tumors improve their survival. These observations correlate with 5-FU treatment of MMR-deficient cells in that these cells are resistant to 5-FU [7], [8], [9], and continue to survive in its presence.

The DNA MMR system plays an important role in maintaining DNA fidelity after DNA synthesis for cell replication. DNA MMR has two recognition complexes for DNA alterations. hMutSα, a heterodimer of the MMR proteins hMSH2 and hMSH6, recognizes base-base and insertion/deletion (I/D) loops less than two nucleotides [10], whereas ID loops more than 2 nucleotides are recognized by hMutSß, an hMSH2-hMSH3 heterodimer [11], [12], [13], [14], [15], [16], [17], [18]. Notably, hMutSα not only recognizes a nucleotide mispair, but can also recognize altered nucleotides that are intercalated or formed with chemotherapy, such as the adduct O^6^-methylguainine, and intrastrand crosslinking induced by cisplatin [19]. We and others have further demonstrated that 5-FU incorporated into DNA is recognized by hMutSα [9], [20], [21]. Systemic 5-FU therapy leads to incorporation into all forms of RNA, but by its action upon thymidylate synthetase (TS), 5-FU after conversion to a deoxyribonucleic acid serves as a substrate for DNA synthesis with cell depletion of TTPs. It has been estimated that as much as 10% of cellular 5-FU is incorporated into DNA where MMR can recognize, bind, and signal cell death [7]. Isolation of 5-FU in DNA specifically triggered a DNA MMR-dependent cell death [22]. In the absence of DNA MMR, these events do not occur, and account for the cell resistance and lack of survival improvement for patients with MMR-deficient tumors. Because of some indications in the literature regarding hMSH3, a component of hMutSß, in participating in the repair of psoralen and platinum compounds [23], [24], we wondered if hMutSß could recognize 5-FU. Given that 5-FU incorporated into DNA would best simulate a single mispair, we initially predicted that hMutSß would not bind or recognize 5-FU, unlike hMutSα. The presence of hMSH3, the DNA recognition component of hMutSß, is the likely molecule that prevents the occurrence of elevated microsatellite alterations at selected tetranucleotide repeats (EMAST) in colorectal cancers, as reduced expression of hMSH3 has been detected among these tumors [25], [26]. EMAST (and hMSH3 deficiency) is associated with CRC progression, advanced staged tumors, poor prognosis, and African American race [27]. hMutSß has not been previously assessed for recognition of 5-FU incorporated into DNA.

Here, we purified the hMutSß complex as a heterodimers of hMSH3 and hMSH2 and examined its binding ability for 5-FU that is incorporated into DNA. Overall, we herein show that 5-FU cell toxicity correlates directly with relative hMutSα and/or hMutSß binding ability.

## Results

### The level of 5-FU cytotoxicity depends upon the hMutSα and hMutSß status of the cell

**Figure 1:**
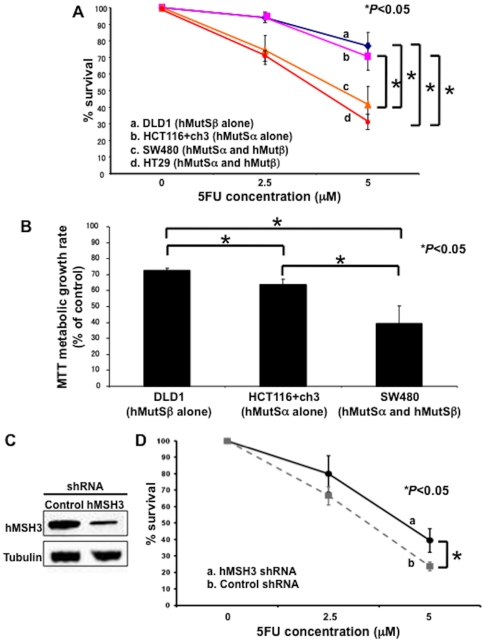
Cell survival after 5-FU treatment is influenced by the hMutSα and hMutSß status. (**A**) Clonogenic assay of hMutSα and/or hMutSß proficient cells in response to 5-FU. Cells were plated in media containing 0, 2.5, 5 µM 5FU and allowed to form colonies over 10 days. The plates were then fixed with methanol and stained with 3% Giemsa, and viable colonies were counted. (**B**) MTT assay of the same cells as above, with mean values shown with standard deviations. Each cell line was incubated with the 5 µM 5FU for 2.5 and 5 days. (**C, D**) SW480 were stably transfected with an hMSH3 shRNA expression plasmid for hMSH3 knockdown, and stable hMSH3-deficient clones were utilized for clonogenic assay in media containing 0, 2.5, 5 µM 5-FU and allowed to form colonies over 20 days (**D**). The plates were then fixed with methanol and stained with 3% Giemsa, and viable colonies were counted. Western blotting with anti-hMSH3 antibody was performed to confirm the hMSH3 knockdown (**C**). Each experiment was performed in triplicate, and the experiment replicated three independent times. * = P<0.05.

We performed two growth assays to identify if hMutSß had any contribution towards 5FU cytotoxicity. By clonogenic assay, the number of colonies in hMutSα/hMutSß double competent cells was smaller than that in either hMutSα alone competent cells (P<0.05) or hMutSß alone competent cells (P<0.05) (**Fig. 1A**). The results of clonogenic assays were confirmed by MTT assay (**Fig. 1B**). In addition, the results utilized by MTT assay showed that hMutSα/hMutSß double competent cells had the lowest survival rate (P<0.05), followed by intermediate survival in hMutSα alone positive cells (P<0.05) and the best survival in hMutSß alone positive cells (P<0.05) (**Fig. 1B**). To further clarify the role of hMutSß for 5FU cytotoxicity, we transfected SW480 (MMR-proficient colorectal cancer cells) with an hMSH3 shRNA expression plasmid (**Fig. 1C**). Knock down of hMSH3 reversed some 5-FU-induced cell death over control cells by clonogenic assay (**Fig. 1D**). These results indicate that not only hMutSα triggers 5-FU cytotoxicity as reported previously [9], [20], [21], but hMutSß also contributes to the 5-FU cytotoxicity. The contribution towards 5-FU cytotoxicity appears greatest when both hMutSα and hMutSß are present, with both completes contributing a portion towards cell death.

### Purified hMutSß recognizes and binds 5-FU-incorporated into DNA, and exhibits ATP-induced dissociation

**Figure 2:**
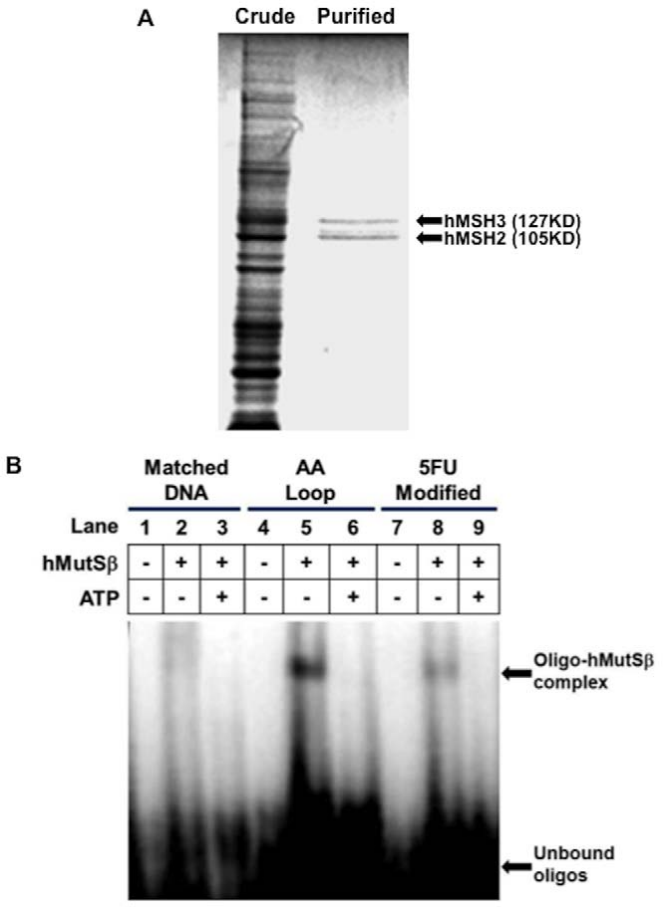
Purification of hMSH3-hMSH2 heterodimer, and binding of hMutSß to 5-FU incorporated within DNA. (**A**) Purity of baculovirus-synthesized hMutSβ on Coomassie blue staining of electrophoresed protein extracts. Extracts from crude baculovirus-synthesized proteins (left lane), and FPLC-purified baculovirus-synthesized proteins (right lane) were electrophoresed on a 7.5% PAGE gel, and stained with 0.1% Coomassie blue. Note the retention of 127 kD and 105 kD protein bands in FPLC-purified extracts, which by Western blotting corresponded to hMSH3 and hMSH2 proteins. (**B**) Electromobility gel shift assays utilizing purified hMutSß and complementary (lanes **1–3**), AA I/D loop (lanes **4–6**), and 5-FU-containing oligonucleotides (lanes **7–9**). Radiolabeled oligonucleotides were incubated with or without purified hMutSß in the presence or absence of 4 mM ATP. Note the acquisition of an oligo-hMutSß band with the two adenines ID loop (lane **5**) or 5-FU-containing DNA (lane **8**) when incubated with purified hMutSß, and the dissolution of the complex with the addition of ATP (lane **6**, lane **9**). Intensity of the band formed between 5-FU-modified DNA and hMutSß was weaker than that of the AA I/D loop formed with hMutSß.

Based on the cell growth data that indicates a contribution of hMutSß for 5-FU cytotoxicity, we investigated the hMutSß recognition of 5-FU incorporated into DNA. We successfully purified a stable recombinant hMutSß complex (**Fig. 2A**) similar to our previously described approach for the hMutSα complex [20]. We co-infected Sf9 cells with hMSH2 and hMSH3 baculoviral constructs, and extracted and purified the proteins as a heterodimer utilizing two separate FPLC purification columns. Coomassie blue staining verified hMutSß purification (**Fig. 2A**), which we further confirmed was a heterodimer by immunoprecipitation of purified hMutSß with hMSH2 and hMSH3 antibodies (not shown). Utilizing the purified hMutSß for EMSA, we examined hMutSß ability to bind perfect complementary DNA, an AA insertion/deletion (I/D) loop, as well as 5-FU-modified DNA. As shown in **Fig. 2B**, purified hMutSß demonstrated little binding to complementary DNA, whereas significantly more binding was observed with the AA I/D loop (**Fig. 2B**). Interestingly, EMSA demonstrated that purified hMutSß bound 5FdU-modified DNA (**Fig. 2B** **, lane 8**) but to a lesser degree than hMutSß binds DNA containing the AA I/D loop (**Fig. 2B** **, lane 5**). Controls without protein did not show the hMutSß-DNA complex band. The addition of ATP causes hMutSß to dissociate from DNA. We demonstrate that purified hMutSß disassociates from DNA containing 5-FU as well as from DNA containing the AA I/D loop when 4 mM ATP was added to the hMutSß/DNA mixture (**Fig. 2B** **, lanes 3, 6, and 9**). This is analogous to our observation for ATP-dependent dissociation of hMutSα from DNA containing 5FdU [20].

### Purified hMutSß dynamically binds 5-FU in DNA, but hMutSα binds to 5-FU to greater extent than hMutSß

**Figure 3:**
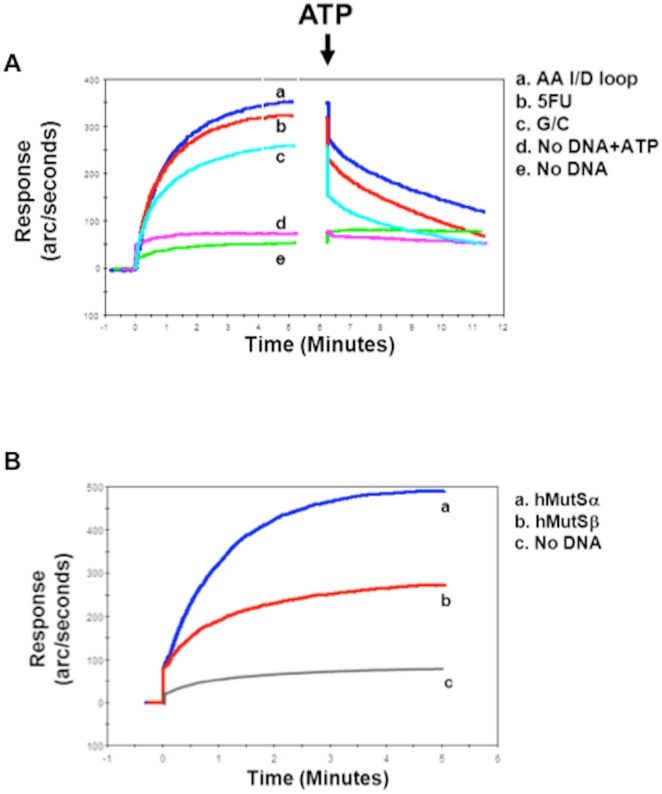
Dynamics of binding and dissociation between hMutSß and 5-FU. (**A**) Biosensor analysis (surface plasmon resonance) of the association (left curves) and ATP-induced dissociation (right curves) between hMutSß and AA I/D loop (curve **a**), 5-FU-containig DNA (curve **b**), or complementary DNA (curve **c**). A dissociation buffer containing 4 mM ATP was added (arrow) after 5 minutes of association for dissolution experiments. Note the initial rapid dissociation upon the addition of ATP, followed by a slower dissolution phase lasting more than 5 minutes to reach steady state for the three DNA substrates. Controls (curves **d** and **e**) indicate the specificity of ATP's effect when hMutSß is bound to a DNA substrate compared to when the DNA substrate is absent. (**B**) Binding affinity of hMutSα (curves **a**) or hMutSß (curves **b**) to 5-FU-containing DNA. hMutSα bound 5-FU DNA ∼2-fold higher than hMutSß in the consecutive experiments.

As we have already demonstrated the binding ability of hMutSα [20] we further examined the dynamics of the binding and dissociation between hMutSß and 5-FU incorporated into DNA to understand the contribution of each of hMutSα and hMutSß for 5-FU recognition. Utilizing the IAsys biosensor system [28], there is essentially no binding of purified hMutSß on IAsys cuvettes without any substrate. In contrast, hMutSß binds to complementary DNA to a low extent. The binding ability of purified hMutSß for 5-FU incorporated into DNA was greater than for perfect complementary DNA (∼1.3-fold increased) but less than for DNA containing the AA I/D loop, which was ∼1.8-fold increased over perfect complementary DNA (**Fig. 3A**). The addition of ATP caused a rapid partial dissociation of hMutSß from all of the DNA substrates and approached steady-state equilibrium 5 minutes after ATP was added with dissociation from the G/C substrate approaching equilibrium faster than dissociation from 5-FU or the AA I/D loop (**Fig. 3A**). To assess the relative binding of hMutSα and hMutSß, we compared affinity levels between hMutSα and hMutSß upon the 5-FU-modified DNA utilizing IAsysh. MutSα binds 5-FU at a greater extent than hMutSß (∼2-Fold increase) (**Fig. 3B**). Our observations demonstrate that hMutSα and hMutSß have separate but also additive roles for recognition of 5-FU incorporated into DNA, which corresponds to the relative cell toxicity triggered by 5-FU incorporated into DNA when the MMR complexes are present.

## Discussion

It has been recently demonstrated that hMSH3, the recognition component of hMutSß, plays an important role in recognizing DNA damage, particularly interstrand crosslinks [23], [24]. Although the importance of this component of hMutSß has been recognized in furthering CRC progression, there is no data regarding hMutSß contributions in executing 5-FU toxicity, a key issue since all major therapy for CRC involves 5-FU. Our study demonstrates (a) that 5-FU cytotoxicity depends on the hMutSα and hMutSß status of cells, with the most cytotoxicity observed when both hMutSα and hMutSß are present, intermediate 5-FU cytotoxicity when hMutSα is retained but hMutSß is deficient, and low 5-FU cytotoxicity when cells are hMutSα deficient but hMutSß proficient, (b) that hMutSß recognizes 5-FU-containing DNA, (c) and that the binding affinity of hMutSß is lower than that of hMutSα, which directly correlates with 5-FU cytotoxicity. This is the first study demonstrating an additive role for 5-FU cytotoxicity for hMutSα and hMutSß.

Our data indicate that 5-FU cytotoxicity is more severe in cells that retain both hMutSα and hMutSß than the cells that lack hMutSα and retain hMutSß, which support our prior data that hMutSα recognition of 5-FU incorporated into DNA plays an important role for 5-FU chemosensitivity [20]. It is somewhat surprising that our EMSA results showed that a single base-pair of 5-FU incorporated into DNA is recognized by hMutSß in spite of its general role for recognition of I/D loops greater than 2 molecules, and not single mispairs. This could be due to something unique regarding 5-FU, such as its negative charge, or an atypical or unrecognized role for hMutSß in recognizing altered nucleotides. Takahashi *et al*. demonstrated that cells that lack *hMSH3*, a component of hMutSß, are more sensitive to cysplatin and oxaliplatin than *hMSH3*-proficient cells [24]. Interestingly, they demonstrated that the difference of chemosensitivity between *hMSH3*-deficient and *hMSH3*-proficient cells occurred independently of *hMLH1* status. In addition, it has been shown that hMutSß recognizes ICLs induced by psoralen [23], and is not dependent on hMutSα or hMLH1. hMutSß was also reported to interact with nucleotide excision repair (NER) proteins, and homologous recombination (HR) repair level for ICLs is also dependent on hMutSß and not on hMutSα, which may suggest that hMutSß may cooperate with the NER or HR proteins for ICLs repair perhaps independently of traditional DNA MMR [29]. It is possible that hMutSß recognition of 5-FU incorporated into DNA might trigger MMR-independent repair mechanisms on top of classical DNA MMR [8], [9], [30].

Our data indicates a direct relationship of MMR complex affinity for binding and 5-FU cytotoxicity. Using surface plasmon resonance (IAsys), we measured a 2- fold difference in binding between hMutSα and hMutSß, with hMutSα having the greater affinity for 5-FU within DNA. In addition, the results of IAsys and cell growth assays suggest that hMutSα and hMutSß have additive roles in triggering 5-FU cytotoxicity. Overall, our functional observations of hMutSß in addition to hMutSα [20] for 5-FU cytotoxicity suggest it important to understand a patient's tumor character and functional genotype.

A number of studies indicate that patients with advanced colorectal cancer that DNA MMR-deficient do not derive a survival benefit with systemic 5-FU chemotherapy. The majority of these studies contained patients with somatic hypermethylation of the *hMLH1* promoter, rendering the tumor completely MMR-deficient because for repair to occur after DNA synthesis, both recognition of mismatch in the DNA by either hMutSα or hMutSß and signaling for excision of the mismatch by a second heterodimer, hMutLα (consisting of the MMR proteins hMLH1 and hPMS2), are essential. In contrast, partial MMR function remains when either hMutSα (normal function of both hMutSß and hMutLα) or hMutSß (normal function of hMutSα and hMutLα) is lost. There are at least two scenarios for which hMutSα and/or hMutSß function might be exploited for a partial response to 5-FU. The first is Lynch syndrome patients with an *hMSH6* germline mutation (as opposed to hMLH1 or hMSH2 mutations, and there has not been any *hMSH3* germline mutations identified to date). These patients might demonstrate partial response to 5-FU when compared to Lynch syndrome patients with *hMLH1* or *hMSH2* germline mutations. The second is with patients whose tumors show EMAST. Up to 60% of colon and 30% of rectal cancers have EMAST, and EMAST is associated with loss of hMSH3 expression. Patients with EMAST tumors might demonstrate a reduced 5-FU response compared to patients with MSS tumors, and improved response when compared to patients with hMLH1 hypermethylation in their tumors.

In conclusion, we demonstrated the hMutSß recognition of 5-FU incorporated into DNA, and both hMutSα and hMutSß has additive roles in triggering 5-FU cytotoxicity. Our data indicates that the MMR complexes provide a hierarchical chemosensitivity for 5-FU cell death. Our findings may have implication for specific Lynch syndrome patients as well as specific CRC patients whose tumors demonstrate EMAST. These groups of patients should be studied for their response to 5-FU systemic therapy.

## Materials and Methods

### Cell Lines, Cell Culture and Transfection

The human colon cancer cell lines DLD1, HCT116+ch3, HT29 and SW480 were obtained from American Type Culture Collection (Rockville, MD) and maintained in growth medium containing 10% fetal bovine serum (FBS). Of these cell lines, DLD1 is defective for hMutSα (hMutSß competent), HCT116+ch3 is deficient for hMutSß (hMutSα competent), and SW480 and HT29 cells have been described proficient in and stable at microsatellites (hMutSα and hMutSß competent). For isolation of stable hMSH3-deficient clones, SW480 cells were transfected with a retroviral vector that encodes shRNA to hMSH3 (kind gift by the C. Richard Boland laboratory [24]) by using FuGENE®6 (Roche, IN), and selection was done using both 400 µg/ml of G418 and 1 µg/ml of puromycin. After selection, colonies were pooled and cultured for analysis. Stable cell lines were confirmed by DNA sequencing.

### Reagents

5-FU was obtained from Sigma Chemical Co. (St. Louis, MO) and dissolved in Iscove's modified Dulbecco's medium at a stock concentration of 1 mmol/L and maintained at 4°C.

### Clonogenic assay

Exponentially growing cells were trypsinized and washed twice with PBS. Cells were then plated on 60×15 mm Tissue Culture Dish (Becton Dickinson Labware, NJ) in Iscove's modified Dulbecco's medium supplemented with 10% FBS and containing various concentrations of 5-FU (0, 2.5, and 5 µmol/L), then incubated at 37°C and 5% CO_2_. After 10 days of growth, the culture plates were washed with PBS, fixed with methanol for 15 minutes, and then rewashed with PBS. The colonies were stained with 3% Giemsa (Sigma, St Louis, MO) for 15 minutes and rinsed with water. Previously viable clonal colonies of at least 50 cells were counted. The relative surviving fraction for each cell line was expressed as a ratio of the plating efficiency in treated cultures to that observed in the controls.

### 3-(4,5-dimethylthiazol-2-yl)-2,5-diphenyltetrazolium bromide (MTT) assay

Exponentially growing cells were seeded onto 96-well plates and grown for 1 day. Cells were treated with or without 5-FU at the concentration of 5 µM. After 2.5 and 5 days later, 5 mg/ml MTT (Sigma, St Louis, MO) in PBS was added and incubated for 3 h. The absorbance was determined at 570 nm with a microplate reader (Biorad).

### Synthesis of 5-FU-containing oligonucleotides

We synthesized 38-mers in an oligonucleotide synthesizer (ABI, Foster City, CA) at the UCSD Cancer Center Oligonucleotide Synthesis Core for the experiments. For a negative control (i.e. perfect complement), we made 5′-TTTCTGACTTGGATACCATCTATCTATCTATAAAATAT-3′ and for a positive control, we made 5′-TTTCTGACTTGGATACCATCTATCTATCTATAA-**AA-**AATAT-3′ which differs from the perfect complement at the 34th nucleotide (insertion of two adenines). To synthesize the 5-FU-containing DNA, we utilized the phosphoramdite form of 5-fluorodeoxyuracil (5FdU) (Glenn Research, Sterling, VA) to make 5′-TTTCTGACTTGGATACCA **-5FdU-** CTATCTATCTATAAAATAT-3′. To complete the double-stranded DNA molecule, the complementary sequence of the 38-mer was synthesized (5′-ATATTTTATAGATAGATAGATGGTATCCAAGTCAGAAA-3′), end-labeled with ^32^P, and equal molar ratios of the 38-mer containing the 5-FU, the AA ID loop, or unaltered strand and the complementary strand was mixed, heated to 95°C, and allowed to cool slowly.

### Production and purification of the recombinant hMutSα and hMutSß Protein

*Spodoptera frugiperda 9 (Sf9)* cells (typically 1.2×10^8^) were co-infected with a mixture of hMSH2 and hMSH3 recombinant baculoviruses constructs (gift of Josef Jiricny, Ph.D. and Giancarlo Marra, Ph.D.) at a multiplicity of infection of 10. After 72 h, the cells were collected and total protein extracts were prepared. The extracts were sedimented at 20,000 *g*, and the supernatant was then diluted with buffer A (25 mM HEPES/NaOH, pH 7.6, 1 mM EDTA, 2 mM ß-mercaptoethanol), and loaded onto a 5-ml Hi-Trap Heparin-Sepharose fast protein liquid chromatography (FPLC) column (Amersham Pharmacia Biotech, Arlington Heights, IL). The protein complex was eluted with a 45 ml linear gradient from 25 to 100% of buffer B (25 mM HEPES/NaOH, pH 7.6, 1 M NaCl, 1 mM EDTA, 2 mM ß-mercaptoethanol). The fractions containing the hMutSß heterodimer were determined by Western blot and pooled, diluted with buffer A to a conductivity corresponding to 15% salt, and loaded onto a 5-ml HiTrap Q FPLC column (Amersham Pharmacia Biotech, Arlington Heights, IL). The fractions containing the pure hMutSß complex (eluting at around 550 mM NaCl) were pooled and stored in aliquots at −80°C. We assessed purity of the recombinant proteins through Coomassie staining (0.1%) of 7.5% PAGE gels electrophoresed with the eluted proteins. hMutSα was produced and purified as the same manner as previously reported [20].

### Electromobility gel shift assays (EMSA)

EMSAs were performed as described previously [10], [20]. Complementary DNA was labeled at their 5′ ends by using T4 polynucleotide kinase and [γ-^32^P] ATP, and this single DNA strand was annealed to the three synthesized 38-mer strands. Binding reactions contained the purified hMutSß (100 nM), and 1×10^6^ cpm of ^32^P-labeled double-stranded DNA substrates in a final volume of 10 µl of binding buffer (20 mM HEPES pH 7.6, 1 mM EDTA, 1 mM DTT, and 10% glycerol (v/v), 100 µg of poly (dI-dC)). DNA protein complexes were separated under non-denaturing conditions on a 6% polyacrylamide gel using 1× TBE (89 mM Tris borate, 89 mM boric acid, 2 mM EDTA) as a running buffer. The gels were then dried, and protein-DNA complexes were visualized using a phosphorimager (Molecular Dynamics, Sunnyvale, CA). To determine specificity of binding of hMutSß to the DNA, 4 mM ATP was added to release the DNA-protein complexes.

### DNA binding analysis (IAsys)

The binding and dissociation characteristics between the MMR complex and DNA substrates was performed as described previously [20], [28]. We utilized the IAsys biosensor system (Affinity Sensors, Cambridge, UK) to characterize the hMutSß-DNA interaction. The IAsys system utilizes surface plasmon resonance to detect total internal reflectance measurements. We synthesized a 5′-biotinylated DNA complementary oligomer (see sequence above) and annealed this to each of the three 38-mers to make double-strand DNA to bind the IAsys streptavidin-coated SA sensor chip (IAsys Auto Plus microcuvettes, Affinity Sensors). The protein heterodimer hMutSß was diluted to 100 nM in running buffer (0.02 M NaCl, 25 mM Tris-Cl, pH 7.8, 1 mM DTT, 2%glycerol, 0.05% IGEPAL 20 and 10 mM MgCl_2_) and added to the chip for monitoring and recording the interaction for 5 minutes. Dissociation studies were performed by adding dissociation buffer (containing 4 mM ATP) for 5 minutes. Two washes of 50 µl 3 M NaCl were used to regenerate the binding surface of the microcuvette after each injection of hMutSß. All experiments were performed at 25°C. Data were collected and analyzed using the IAsys evaluation software (IAsys plot version 3.0).
